# Fine-scale spatial and temporal variation of clinical malaria incidence and associated factors in children in rural Malawi: a longitudinal study

**DOI:** 10.1186/s13071-018-2730-y

**Published:** 2018-03-05

**Authors:** Alinune N. Kabaghe, Michael G. Chipeta, Steve Gowelo, Monicah Mburu, Zinenani Truwah, Robert S. McCann, Michèle van Vugt, Martin P. Grobusch, Kamija S. Phiri

**Affiliations:** 1 Center of Tropical Medicine and Travel Medicine, Department of Infectious Diseases, Academic Medical Center, University of Amsterdam, 1105 AZ Amsterdam, Netherlands; 2 School of Public Health and Family Medicine, College of Medicine, University of Malawi, Blantyre 3, Malawi; 3 Lancaster University, Lancaster Medical School, Lancaster, LA1 4YG UK; 4 Malawi-Liverpool Wellcome Trust, P O Box 30096, Blantyre 3, Malawi; 5 Laboratory of Entomology, Wageningen University and Research, 6708 PB Wageningen, Netherlands; 6 Management Sciences for Health - Malawi Program, EBC Building, Off Paul Kagame Road, Private Bag 398, Lilongwe 3, Malawi

**Keywords:** Malaria, Spatio-temporal heterogeneity, Incidence rate, Entomological surveillance

## Abstract

**Background:**

Spatio-temporal variations in malaria burden are currently complex and costly to measure, but are important for decision-making. We measured the spatio-temporal variation of clinical malaria incidence at a fine scale in a cohort of children under five in an endemic area in rural Chikhwawa, Malawi, determined associated factors, and monitored adult mosquito abundance.

**Methods:**

We followed-up 285 children aged 6–48 months with recorded geolocations, who were sampled in a rolling malaria indicator survey, for one year (2015–2016). Guardians were requested to take the children to a nearby health facility whenever ill, where health facility personnel were trained to record malaria test results and temperature on the child’s sick-visit card; artemisinin-based combination therapy was provided if indicated. The cards were collected and replaced 2-monthly. Adult mosquitoes were collected from 2-monthly household surveys using a Suna trap. The head/thorax of adult *Anopheles* females were tested for presence of *Plasmodium* DNA. Binomial logistic regression and geospatial modelling were performed to determine predictors of and to spatially predict clinical malaria incidence, respectively.

**Results:**

Two hundred eighty two children, with complete results, and 267.8 child-years follow-up time were included in the analysis. The incidence rate of clinical malaria was 1.2 cases per child-year at risk; 57.1% of the children had at least one clinical malaria case during follow-up. Geographical groups of households where children experienced repeated malaria infections overlapped with high mosquito densities and high entomological inoculation rate locations.

**Conclusions:**

Repeated malaria infections within household groups account for the majority of cases and signify uneven distribution of malaria risk within a small geographical area.

**Electronic supplementary material:**

The online version of this article (10.1186/s13071-018-2730-y) contains supplementary material, which is available to authorized users.

## Background

Spatio-temporal variations in malaria burden are due to individual, household, community and environmental factors [1–5]. There is a need to monitor and identify spatio-temporal variations of malaria burden to inform decision-making; however, measuring these variations is complex and costly. Although studies measuring spatio-temporal variations using longitudinal design exist [6, 7], most studies rely on cross-sectional data from household surveys and health facility records [1]. Cross-sectional surveys can measure temporal changes of malaria if repeated over short durations [8, 9] or use serological markers [10–12], which are expensive and operationally demanding to implement. Health facility data in most sub-Saharan Africa health systems are unreliable, incomplete, depend on access to health care [13–15], and lack the fine spatial scale needed to accurately identify high burden areas.

When incidence of clinical malaria is used for detecting spatial variations, it should only be used in young children who are defined as having low immunity. In high transmission settings, clinical malaria mainly occurs in children below five years of age, before naturally-acquired immunity develops [16, 17]. After repeated exposure, individuals above five years are less likely to develop clinical malaria [16]. Measuring clinical malaria in children, by using age as a proxy to previous exposure, may be useful in monitoring the spatial and temporal patterns of burden in the community. These patterns enable identification of high malaria burden areas to inform decision-making. Prospective cohort studies of clinical malaria incidence in children, reporting high spatial resolution, are unavailable from Malawi and are limited from most high-transmission settings.

We aimed to measure precisely, i.e. with a high geographical resolution, the incidence of clinical malaria, in a cohort of children below five years of age in a rural Malawian community, and to determine associated factors. We also describe the spatio-temporal variation in adult mosquito abundance. The study was done as part of a baseline assessment of malaria burden in Majete Wildlife Reserve in Chikhwawa, Malawi.

## Methods

### Study design and setting

This was a prospective cohort study of malaria incidence in children below five years old in the Majete Wildlife Reserve (MWR) perimeter; recruited children were followed up for 12 months. The children were recruited from households enrolled in the rolling Malaria Indicator Survey (rMIS) [8] between July and October 2015; follow-up of participants ended in November 2016. For adult mosquito sampling, we included data from repeated cross-sectional surveys from July 2015 to August 2016.

The study was done under the umbrella of the Majete Malaria Project (MMP). The study area has been previously described [8, 18]. MWR perimeter is a rural area situated in Chikhwawa District in southern Malawi and lies within the Rift Valley between 60–500 m above sea level. The population in the perimeter rely mainly on rain-fed farming. Malaria transmission occurs throughout the year but peaks during the rainy season spanning November to May. Study participants were from three household-enumerated areas named Focal Area A, B and C. The study area comprised 65 villages, approximately 6600 households and a population of approximately 25,000 people in December 2014. The study area has one district hospital, six health centres and five village clinics providing primary health care. Uncomplicated malaria diagnosis and treatment for children below five years using malaria rapid diagnostic tests - mRDT (SD Bioline malaria Ag Pf HRP-2 Standard Diagnostics Inc, Gyeonggi-do, Republic of Korea) and first line artemether-lumefantrine (AL), respectively, are free in these facilities.

### Cohort study participants and recruitment

Children were eligible to participate if they were aged 6–48 months, permanently resided in the study area, and if their parent or legal guardian consented. At recruitment, children were actively screened for malaria symptoms (reported fever or a temperature of 37.5 °C and above). All recruited participants were given sick-visit cards, which were presented together with their health passport, a mandatory health information document, to the surrounding health facility each time they were unwell.

At recruitment, we recorded the child’s household geolocation using Global Position System on a Samsung Galaxy tablet running the Android 4.1 Jellybean operating system. We also recorded age, sex and household ownership of items including an insecticide-treated bed net (ITN), presence of open eaves in their house and anthropometric measurements. An eave is the point where the roof and wall of the house meet, which may serve as a potential mosquito entry point if open [19, 20]. The children were tested for malaria using an mRDT (SD Bioline malaria Ag Pf as above) and haemoglobin level using Hemocue 301® (Haemocue, Angelholm, Sweden). Children with uncomplicated malaria were prescribed first line treatment (AL); children with a haemoglobin less than 11 g/dl were referred to the nearest health facility.

### Follow-up and data collection

The follow-up of cases was based primarily on passive case detection at the health facilities. All health workers from the surrounding health facilities involved in clinical management of under-five children underwent a one-day study procedures orientation. The health workers were trained to record malaria diagnosis and mRDT results on the sick-visit cards each time a child in the study presented at their facility. If the sick-visit was not recorded in the sick-visit card by the health worker but in the health passport, a research nurse transcribed details from the health passport to the sick-visit card; health workers are required to record details of each clinical consultation in a health passport as part of their standard work.

At two-monthly intervals for 12 months, study personnel visited the households of the participants to collect and replace sick-visit cards. At 6- and 12-month follow-up household visits, the children were screened for malaria symptoms as described above, and only symptomatic children were tested for malaria using an mRDT. If positive, the participant was treated, and this information was recorded on the sick-visit card as a clinical malaria case. After checking records on the sick-visit card with health passport records, the data were entered into a tablet on open data kit platform and sent to a remote server *via* an internet connection.

### Entomological surveys

Sampling of adult mosquitoes was planned for 195 households every two months using Suna traps [21]. At each house, a Suna trap was set for one night indoors and one night outdoors. The sequence of indoors and outdoors was based on a coin toss for each house. All *Anopheles* mosquitoes collected were identified morphologically based on the Gillies & Coetzee key [22] and then by polymerase chain reaction [23, 24]. The head/thorax of female *Anopheles* mosquitoes were separated from the abdomen and tested for the presence of *Plasmodium falciparum* (*Pf*) DNA using quantitative polymerase chain reaction (qPCR) [25, 26].

### Data sources and variables

Clinical malaria was defined as a documented diagnosis of malaria and documented mRDT positive result recorded or transcribed on a sick-visit card during a sick-visit or a 6- or 12-month study visit. For children who had a clinical malaria diagnosis more than once within 14 days, only the first clinical malaria case was included as an outcome.

A HOBO weather station (Onset Computer Corporation, Massachusetts, USA) recorded hourly rainfall in mm; hourly average temperature in degrees Celsius (°C); and hourly relative humidity as a percentage. The weather data were summarised as monthly average temperature, total rainfall and average humidity (data from Focal Area B is reported in this paper). Normalised difference vegetation index (NDVI) data were calculated based on images from the Landsat 8 satellite, downloaded from the United States Geological Survey (USGS; http://earthexplorer.usgs.gov/). Elevation data were derived using the Advanced Space-borne Thermal Emission and Reflection Radiometer Global Digital Elevation Model (ASTER GDEM) version 2, downloaded from USGS (http://gdex.cr.usgs.gov/gdex/).

### Sample size and sampling

A sample size of 285 children was calculated using the following formula: N = (*z*_1 − α/2_/ε)^2^, where z_1-α/2_ is the standard normal deviate for the probability *p* and *ε* is the relative precision. The confidence level was set at 90%, relative precision was 10%; we added 5% of the total N to account for attrition.

All children in three rMIS [8] household sampling rounds were eligible. In each sampling round, 270 houses (90 from each focal area) were sampled using adaptive geostatistical design (AGD) [27] - a probability based sampling method. Due to logistical capacity, mosquito sampling was done in 75% of randomly selected rMIS households. Mosquito sampling required two nights of collection per house, indoors and outdoors.

### Bias

Health system factors, such as differences in availability of diagnostic supplies and health worker practices [28] between health facilities, were a potential source of bias for diagnosing clinical malaria. We attempted to address this source of bias through the health worker training. Sampling bias was avoided by using AGD [27].

### Statistical analysis

We used the statistical software R, version 3.3.1 [29] to analyse the collected data. The main outcome was clinical malaria defined as above. For the overall incidence rate, the time at risk was calculated by subtracting 14 days from the child-years follow-up with each case of clinical malaria treated with AL. Loss to follow-up was assumed to follow the missing completely at random mechanism (MCAR) [30, 31]. Therefore, data from the remaining subjects were assumed to be a random sub-sample of the study population and loss to follow-up leading to observations MCAR should not bias the measures of association and could be ignored in the analyses.

We identified model covariates for the multivariate binomial and geostatistical regression models using forward selection. Normalized difference vegetation index (NDVI) and elevation were selected *a priori* in the geostatistical model. The fitted geostatistical binomial logistic model has the following ingredients: random variables *Y*_*i*_ of positive counts, binomial denominators *m*_*i*_, explanatory variables *d*_*i*_ ∈ *R*^*p*^, and associated sampling locations *x*_*i*_ : *i* = 1, …, *n* in the study region. Conditionally on a zero-mean Gaussian process *S*(*x*) and mutually independent zero-mean Gaussian variable *Z*_*i*_, *Y*_*i*_ follows a binomial distribution with mean *E*{*Y*_*i*_| *S*(*x*_*i*_), *Z*_*i*_} = *m*_*i*_*p*_*i*_ such that

$$ \mathit{\log}\left\{\frac{p}{\left(1-p\right)}\right\}=d{\left({x}_i\right)}^{\prime}\beta +S\left({x}_i\right)+{Z}_i $$

where we set *d*_*i*_ = *d*(*x*_*i*_) to emphasize the spatial context. To generate the prevalence maps, we extracted the values of the covariates at all prediction locations using raster data. We then estimated prevalence, p(x), at a prediction location x as

$$ \widehat{P}(x)=\frac{1}{B}\sum \limits_{i=1}^B\frac{e^{d{(x)}^T\widehat{\beta}+{\widehat{S}}_i(x)}}{1+{e}^{d{(x)}^T\widehat{\beta}+{\widehat{S}}_i(x)}} $$

where ${\widehat{S}}_i(x)$ is the *i*^*th*^ Monte Carlo sample and $\widehat{\beta}$ is the plug-in maximum likelihood estimate. Spatial predictions for clinical malaria were mapped quarterly as Q1 = September to November 2015; Q2 = December 2015 to February 2016; Q3 = March to May 2016; Q4 = June to August 2016.

The entomological inoculation rate (EIR) was calculated using the following formula: Mosquito density × sporozoite rate. Mosquito density was calculated as the number of *Anopheles* females per location per night. Sporozoite rate was calculated as the proportion of female *Anopheles* mosquitoes with *P. falciparum* DNA in the head/thorax.

## Results

### Recruitment and follow-up

**Fig. 1:**
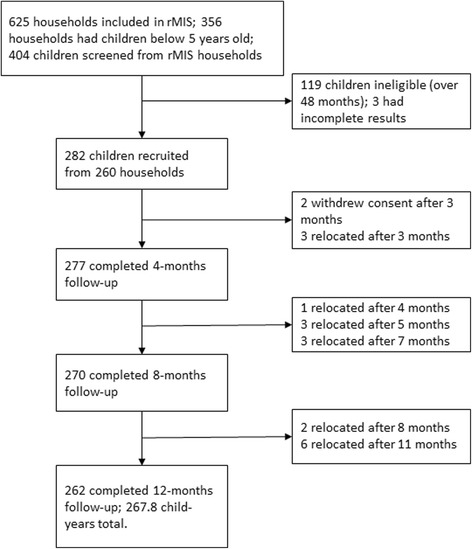
Flow diagram of participants followed-up in the study and the total child-years follow-up completed

Two hundred eighty-five children were recruited between July and October 2015; 265 (93%) children completed the 12 months of follow-up, although only 282 children from 260 households had complete data and are included in the analysis (Fig. 1). From the 260 households, 20 households had two children, one household had three children, and the rest had one child recruited. Most participants who did not complete the follow-up had relocated at different follow-up periods, although guardians for two children withdrew their consent after completing three months of follow-up (Fig. 1). For the incidence rate, we only include 12 months follow-up data for each child.

### Participants’ characteristics

Of the 282 children in the analysis, 37% were aged between 18 and 30 months, and 46.5% were male (Table 1). At recruitment, 43.4% had a positive mRDT and 69.5% had some form of anaemia (Hb < 11 g/dl). Household ownership of at least one ITN was 39%. Most houses were grass-thatched (70.4%) and 39.7% had open eaves.

**Table 1:** Participants and household characteristics at recruitment

	Children, *N* = 282
Age in months: median (IQR)	25.4 (16.8–35.2)
Age categories in months	
6–18 months	78 (27.7)
18–30 months	105 (37.2)
30–48 months	99 (35.1)
Male: *n* (%)	131 (46.5)
Malnourished: *z*-score < -2 SD	
HAZ: *n* (%)	153 (54.3)
WAZ: *n* (%)	74 (26.2)
WHZ: *n* (%)^a^	49 (17.6)
mRDT-positive at recruitment: *n* (%)^a^	122 (43.4)
Haemoglobin in g/dl at recruitment: mean, SD^a^	10.0, 1.7
Anaemic^a^	
None: Hb ≥ 11 g/dl: *n* (%)	86 (30.6)
Mild: Hb 10–10.9 g/dl: *n* (%)	87 (31.0)
Moderate: 7–9.9 g/dl: *n* (%)	89 (31.7)
Severe: < 7 g/dl: *n* (%)	19 (6.8)
Focal area	
A: *n* (%)	106 (37.5)
B: *n* (%)	96 (34.0)
C: *n* (%)	80 (28.4)
Total number of people in household: mean, SD^a^	5.4, 2.0
Wealth score: mean, SD	-0.1, 2.0
Household ownership of at least one ITN: *n* (%)^a^	108 (38.1)
Grass-thatched: *n* (%)	196 (70.4)
Presence of open eaves: *n* (%)^a^	110 (39.7)

*Abbreviations: HAZ* height-for-age z-score, *SD* standard deviation, *WAZ* weight-for-age *z*-score, *WHZ* weight-for-height

^a^Data for at least one child was missing

### Clinical malaria incidence, adult mosquito collections and sporozoite rates

The total duration of follow-up of the participants was 267.8 child-years of which 255.9 were the child-years at risk (Table 2). There were 309 clinical malaria cases recorded of which 9 (2.9%) cases were by active screening. The overall incidence rate of clinical malaria was 1.2 cases per child-year at risk. Out of 282 children, the cumulative incidence, i.e. children with at least one clinical malaria case, was 57.1%. Of the children who had malaria, most had one clinical malaria case (27.7%) and 6.4% had four or more cases during follow-up.

**Table 2:** The number of clinical malaria cases, child-years of follow up and clinical incidence rates by focal area

	Focal area A (*N* = 106)	Focal area B (*N* = 96)	Focal area C (*N* = 80)	Total
Total clinical malaria cases	72	179	58	309
Total follow up child-years	103.3	88.8	75.7	267.8
Total child-years at risk^a^	100.5	81.9	73.5	255.9
Clinical malaria incidence rate: per child-years at risk	0.7	2.2	0.8	1.2
Children with at least one clinical malaria case: *n* (%)	44 (41.5)	75 (78.1)	42 (52.5)	161 (57.1)
One clinical malaria case: *n* (%)	26 (24.5)	23 (24.0)	29 (36.3)	78 (27.7)
2–3 clinical malaria cases: *n* (%)	16 (15.1)	36 (37.5)	13 (16.3)	65 (23.1)
4 or more clinical malaria cases: *n* (%)	2 (1.9)	16 (16.7)	0	18 (6.4)

^a^Calculated from subtracting 14 days from total child-years for each malaria case; child-years at risk also includes the period of follow-up of children who did not complete 12 months

During 1199 indoor and 1211 outdoor collection nights, 351 female anopheline mosquitoes were collected between July 2015 and August 2016; 149 (42.5%) female anopheline mosquitoes were collected indoors (Table 3). Of the female anopheline mosquitoes, 259 (73.8%) were *Anopheles arabiensis*, 78 (22.2%) were *Anopheles funestus* (*s.s.*), 4 were *An. gambiae* (*s.s.*) and 4 were *An. quadriannulatus*. Six specimens identified morphologically as *Anopheles gambiae* (*s.l.*) could not be identified further by PCR. *Plasmodium falciparum* DNA was found in the head/thorax of 32 mosquitoes: 14 in *An. arabiensis*; 15 in *An. funestus* (*s.s.*); 2 in *An. gambiae* (*s.l.*); and 1 in *An. gambiae* (*s.s.*). The overall sporozoite rate was 9.1%; 5.4% in *An. arabiensis* and 19.2% in *An. funestus* (*s.s.*).

**Table 3:** Mosquito species and *Plasmodium falciparum* carriage

Mosquito species	Collected indoors	Collected outdoors	Total no. of mosquitoes	Mosquitoes with Pf sporozoites	Sporozoite rate (%)
*An. arabiensis*	99	160	259	14	5.4
*An. funestus* (*s.s.*)	45	33	78	15	19.2
*An. gambiae* (*s.l.*)	2	4	6	2	33.3
*An. gambiae* (*s.s.*)	2	2	4	1	25.0
*An. quadriannulatus*	1	3	4	0	0.0
Totals	149	202	351	32	9.1

*Abbreviation: Pf Plasmodium falciparum*

### Spatial distribution of clinical malaria incidence, female mosquito density and EIR

**Fig. 2:**
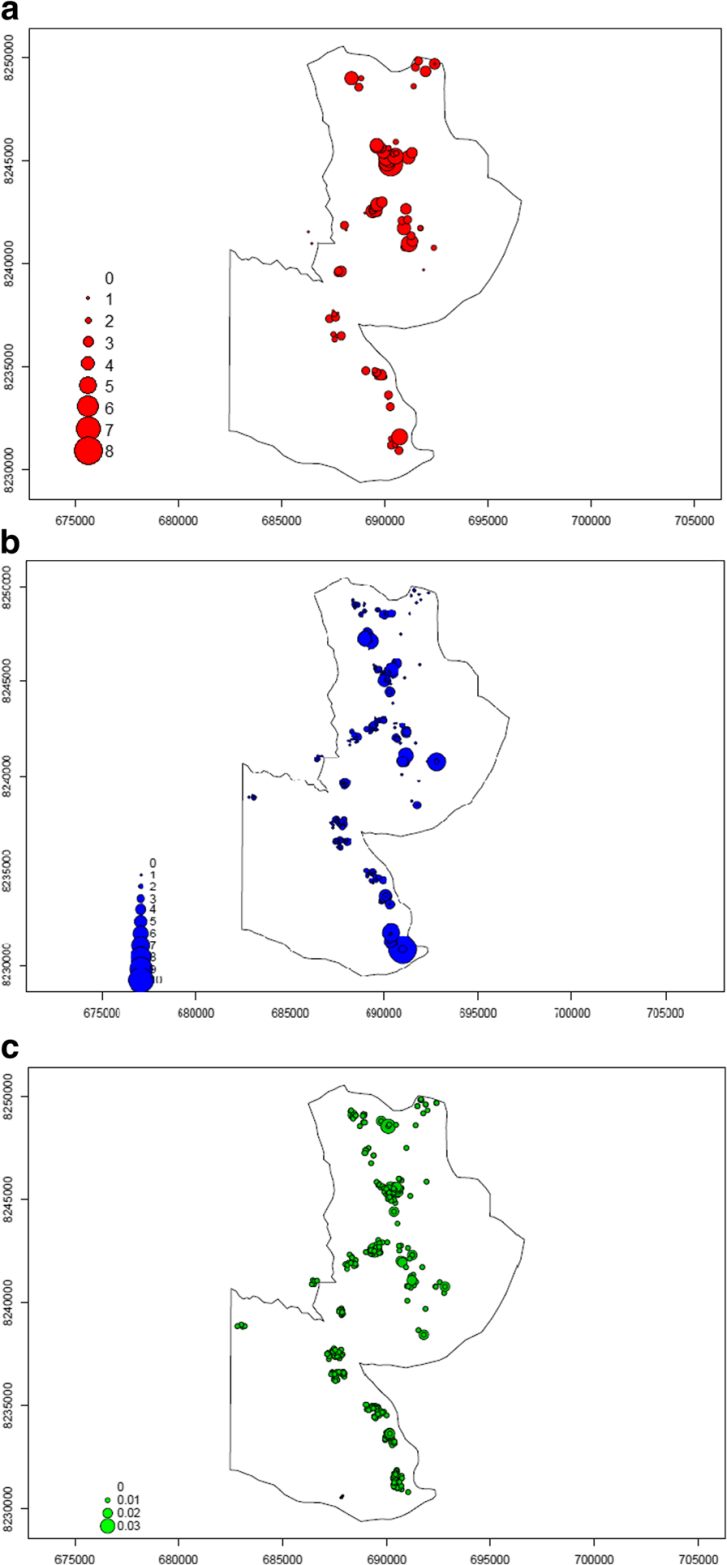
Spatial distribution of clinical malaria incidence, female mosquito density and EIR. The maps show the number of malaria cases (red dots, **a**) occurring per child with the location of the child in Focal Area B. Blue dots (**b**) represent the number of female *Anopheles* mosquitoes per location per night; green dots (**c**) represent the number of infectious female *Anopheles* mosquitoes per location per night (entomological inoculation rate)

We report findings for the spatial distribution of clinical malaria cases, female *Anopheles* mosquito density, and EIR for Focal Area B. Repeated malaria cases occurred in households which were geographically close to each other (Fig. 2a). Some children recorded up to 8 cases of clinical malaria while others had 1 or no infection. High mosquito densities (Fig. 2b) and high EIR locations (Fig. 2c) overlapped the areas of repeated malaria infections.

### Temporal changes in weather and malaria incidence

**Fig. 3:**
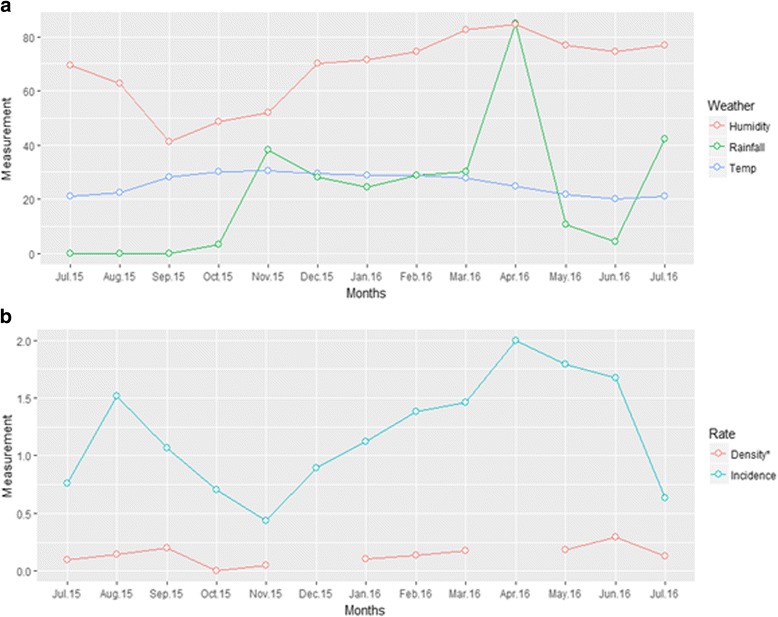
Temporal changes in weather and malaria incidence. The graph shows: **a** variations in monthly rainfall in mm, temperature in °C and relative humidity as percent; **b** mosquito density, and clinical malaria incidence. The incidence peaks during the rainy season with associated increase in humidity. Only weather data from Focal Area B is included

As shown in Fig. 3, malaria incidence rate peaked initially in August 2015 (1.5 clinical malaria cases/child-years) before rapidly declining to the lowest value in November 2015 (0.4 clinical malaria cases/child-years). The second peak of the incidence rate (2.0 clinical malaria cases/child-years) occurred in April 2016, coinciding with the highest recorded rainfall and humidity. This peak results from a gradual rise of the incidence rate, which started in November 2015, and coincided with the beginning of the rains and a rising relative humidity. The temperature varied between 21–30 °C.

### Bivariate and multivariate predictors of clinical malaria incidence

In the bivariate analyses, household ITN ownership and wealth score were associated with a decrease in malaria incidence, while a positive mRDT result at recruitment, presence of open eaves and Focal Area B were associated with an increase. In the multivariate binomial logistic regression model, only Focal Area and wealth score were predictors of malaria incidence overall (Table 4). Increasing wealth score was associated with decreasing odds of malaria incidence (0.96, 0.93–1.00: *P*-value 0.0309). Increasing age was marginally associated with a decrease in the odds of malaria incidence (0.92, 0.80–1.05; *P*-value 0.054).

**Table 4:** Overall bivariate and multivariate odds ratios of clinical malaria

	Unadjusted odds ratio	95%CI	Adjusted odds ratio	95% CI	*P* value*
Negative-mRDT at recruitment	Ref	–	Ref	–	
Positive-mRDT at recruitment	**1.12**	**1.00–1.26**	0.98	0.86–1.10	0.719
6–18 months	Ref	–	Ref	–	
18–30 months	0.98	0.84–1.13	0.92	0.80–1.05	0.054**
30–48 months	1.01	0.87–1.17	0.94	0.81–1.08	0.078**
No ITN in household	Ref	–	Ref	–	
At least one household ITN	**0.88**	**0.78–0.99**	0.92	0.82–1.04	0.202
House with closed eaves	Ref	–	Ref	–	
House with open eaves	**1.17**	**1.04–1.31**	1.02	0.90–1.15	0.792
Wealth score	**0.97**	**0.94–1.00**	**0.96**	**0.93–1.00**	**0.0309**
Focal area A	Ref	–	Ref	–	
Focal area B	**1.44**	**1.27–1.64**	**1.48**	**1.28–1.72**	**< 0.001**
Focal area C	1.14	0.97–1.28	1.13	1.00–1.32	0.070**

*Abbreviations: ITN* insecticide-treated bed net, *mRDT* malaria rapid diagnostic test

*P-value for multivariate model. In bold are variables which are associated with clinical malaria at alpha = 0.05

**P-values which are significant at alpha = 0.1

### Spatial and temporal malaria prediction

**Fig. 4:**
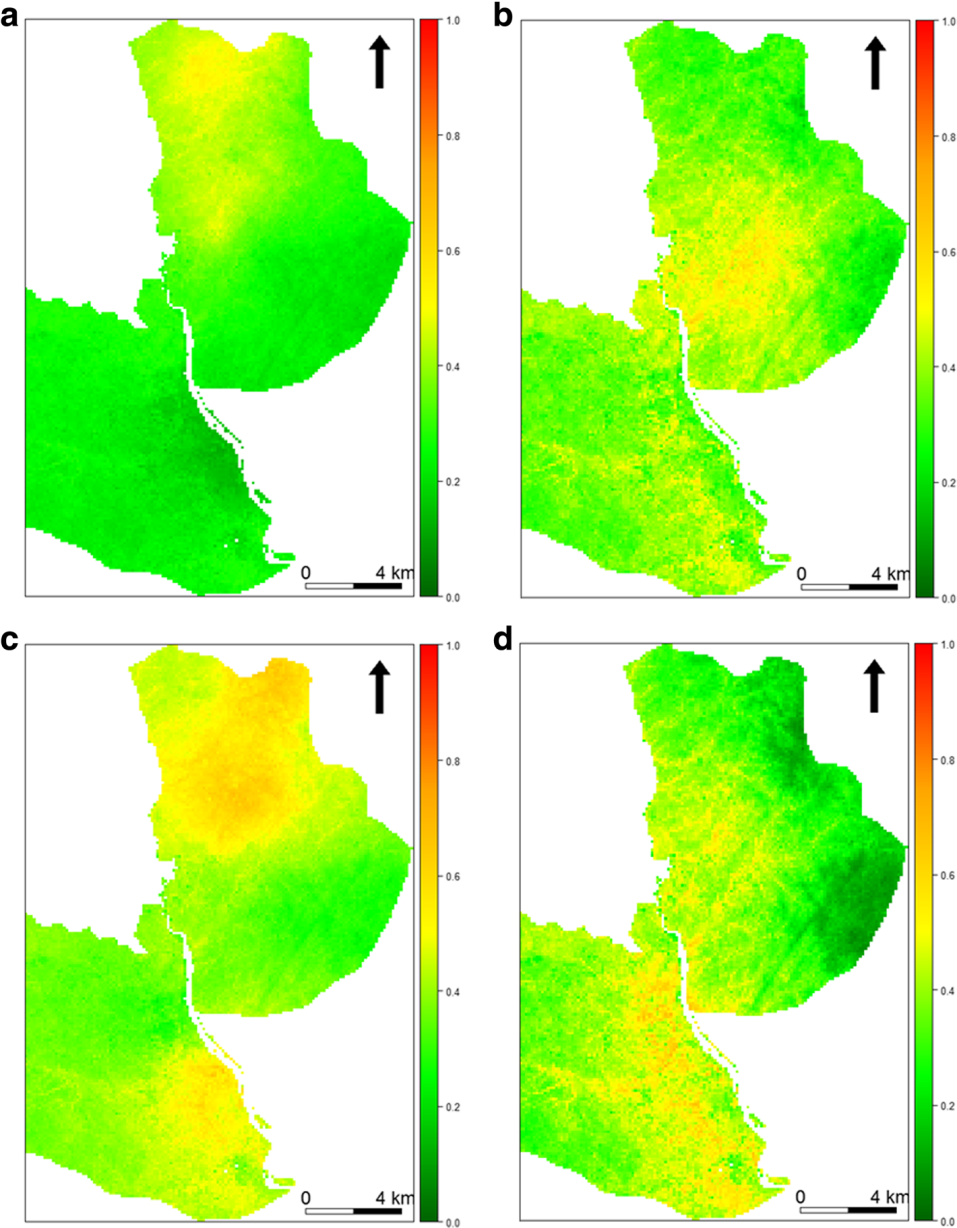
The distribution of malaria incidence by quarter in Focal Area B. **a** Q1: September to November 2015. **b** Q2: December 2015 to February 2016. **c** Q3: March to May 2016. **d** Q4: June to August 2016

Here, we present results from Focal Area B; maps for Focal Areas A and C are provided in Additional files 1 and 2, respectively. The incidence of clinical malaria exhibited marked spatio-temporal variations in Focal Area B (Fig. 4). Predicted clinical malaria incidence was relatively low in Q1 and Q2 and increased in Q3 and Q4. In Q1, before rainfall, the predicted risk of clinical malaria was high (40–50%) only in the northern part of Focal Area B. The risk started to spread widely in Q2 during the rains and by Q3, towards the end of and after the rains, the risk had spread more widely than before and had increased to about 70–80% in certain areas. The predicted risk started to reduce in intensity and coverage in Q4, during the dry season.

## Discussion

To our knowledge, this is the first observational cohort study in children in Malawi reporting fine-scale spatial heterogeneity of clinical malaria incidence. We found marked spatial variations, occurrence of repeated clinical malaria cases, mosquito density and high EIR in households which were close to each other, and notable temporal variations in malaria incidence in children below five years in the MWR perimeter. Results from spatial modelling, malaria distribution mapping, adult mosquito collections, and temporal measurements correlate to highlight where and when malaria cases occurred. We found an overall incidence rate of clinical malaria of 1.2 cases per child-years at risk in this rural, high transmission area. There are no previous reliable estimates of malaria incidence in Malawi, as most studies use health facility-based data [32] rather than prospective observational design.

Clinical malaria was high and unevenly distributed among children between and within the focal areas. Although 42% of children experienced no clinical malaria, 161 children had a total of 309 clinical malaria infections. Repeated infections affect children’s school performance, lead to loss of parental/care takers’ labour time and premature mortality [33, 34]. Repeated infections may also indicate a hotspot where malaria transmission is higher than the surrounding areas [35]. Malaria hotspots have been shown to be dynamic, i.e. change with time, which our models demonstrate. Figures 2 and 4 show the geographical grouping of repeated infections and predicted clinical malaria, respectively, in the same areas. Previous studies in different malaria transmission settings have reported clustering of malaria cases [1, 36, 37]. Geographical groups of households with high mosquito density and EIR overlapped geographically with locations of repeated infections. Vector distribution is more likely related to environmental and household factors in this case. In the current study, predisposing factors such as individual genetics or other household factors were not investigated [38, 39]. To achieve malaria control, an interdisciplinary research approach is required.

Poorer households had a higher risk of malaria, similar to other studies in sub-Saharan Africa [40]. Ettling et al. [41] reported that in Malawi, very low income households spent less on malaria preventive measures and incurred more direct and indirect cost for malaria treatment than other households. Household ownership of ITN at recruitment in this rural community (38%) was lower than the 2014 national aggregated estimate (71%) [42]. With evidence of repeated infections in some households, malaria elimination should be considered a poverty reduction strategy [43]. Prevention of infections will lead to improved productivity and school performance. Malaria reduction has been shown to improve economic growth, and improved social conditions reduce malaria burden [44, 45]. Furthermore, reducing poverty reduces the risk of malaria.

We have also shown temporal variation of clinical malaria in one year of longitudinal data from the quarterly prediction maps and the monthly incidence rates. Clinical malaria was exceptionally high during and after the rainfall and during increased relative humidity, highlighting the importance of weather factors in malaria. Apart from an increase in mosquito larval breeding sites after rainfall in this low-lying area, humidity and (changes in) temperature also affect mosquito larval and malaria parasite development [46, 47]. The scale-up of ITN ownership and use is needed to reduce malaria cases in this rural community. Other vector control strategies, such as larval source management (LSM) [48], should be considered to complement indoor residual spraying and ITN use during the rainy seasons. A community-led LSM strategy is being implemented within this study area to assess feasibility and its effect on EIR [49]. LSM has been shown to reduce the malaria incidence and parasite prevalence in low transmission settings, although it may be challenging to implement in settings where the potential mosquito breeding sites are extensive [50]. Modelling of weather data can also be utilised to forecast malaria burden [51].

In the current study, 39% of households reported the presence of open eaves in their houses, which was independently associated with clinical malaria incidence, similar to other studies [5, 52, 53]. Houses with open eaves are common in rural communities and associated with an increase in malaria vectors compared to houses with closed eaves [19, 54, 55]. House modification by closing open eaves has previously been reported to reduce mosquito entry and anaemia in children [56–58]. Within the study area, a cluster randomised trial is being conducted to assess the effect of community participatory house improvement on malaria prevalence and transmission [49].

Detecting clinical malaria cases in those most at risk and in relation to geospatial distribution, accounting for vector and climate factors, is a vital step towards malaria incidence reduction and transmission interruption. However, such an approach can only precede a more intense multiple-angle coordinated approach when intensified malaria control progresses to elimination. In that case, either focal mass drug administration (MDA) or MDA without pre-testing in previously identified cluster areas in conjunction with targeted mosquito control measures (depending on what would be the most cost-effective and feasible approach in a given setting) should follow. In order to guide such an approach, geospatial detailed work, as depicted here, would be a prerequisite to render any attempt of such an approach cost-effective.

The study had limitations. The use of passive case detection to identify clinical malaria cases relies on access to health services and health-seeking behaviour. Some clinical malaria cases may have been missed if the child got over-the-counter drugs, a health worker did not record a sick-visit in the sick-visit card or health passport (although this is exceptionally rare) or a diagnostic test was not available/recorded. However, by using already existing health workers, the cohort studies can be incorporated and sustained within the health system as a method for monitoring, evaluating and mapping spatio-temporal variations in disease burden. The data presented is only from one year of follow-up. A prolonged data collection period may provide more detailed spatio-temporal patterns.

## Conclusion

Within high transmission areas, there is geographical grouping of clinical malaria and high EIR, where few households are responsible for most of the clinical malaria cases. Less wealthy households and houses with open eaves are at an increased risk of malaria and will require particular attention for malaria control and elimination. Prospective cohort studies may be utilised using passive case detection to monitor disease burden, evaluate effects of interventions and identify high malaria burden areas. These studies can complement national household surveys at sub-district level.

## Additional files

Additional file 1: **Figure S1.** The distribution of malaria incidence by quarter in Focal Area A. **a** Q1: September to November 2015. **b** Q2: December 2015 to February 2016. **c** Q3: March to May 2016. **d** Q4: June to August 2016. (TIFF 340 kb)

Additional file 2: **Figure S2.** The distribution of malaria incidence by quarter in Focal Area C. **a** Q1: September to November 2015. **b** Q2: December 2015 to February 2016. **c** Q3: March to May 2016. **d** Q4: June to August 2016. (TIFF 297 kb)

## Abbreviations

AGD: Adaptive geostatistical design
AL: Artemisinin-lumefantrine
EIR: Entomological inoculation rate
ITN: Insecticide-treated bed nets
MMP: Majete Malaria Project
mRDT: Malaria rapid diagnostic test
MWR: Majete Wildlife Reserve
NDVI: Normalized difference vegetation index
PCR: Polymerase chain reaction
Pf: *Plasmodium falciparum*
rMIS: Rolling malaria indicator survey
