# Supplementary figures and images for: Fine-scale spatial and temporal variation of clinical malaria incidence and associated factors in children in rural Malawi: a longitudinal study
Source: Parasit Vectors. 2018 Mar 5;11:129. doi: 10.1186/s13071-018-2730-y (PMC5839004; doi:10.1186/s13071-018-2730-y)

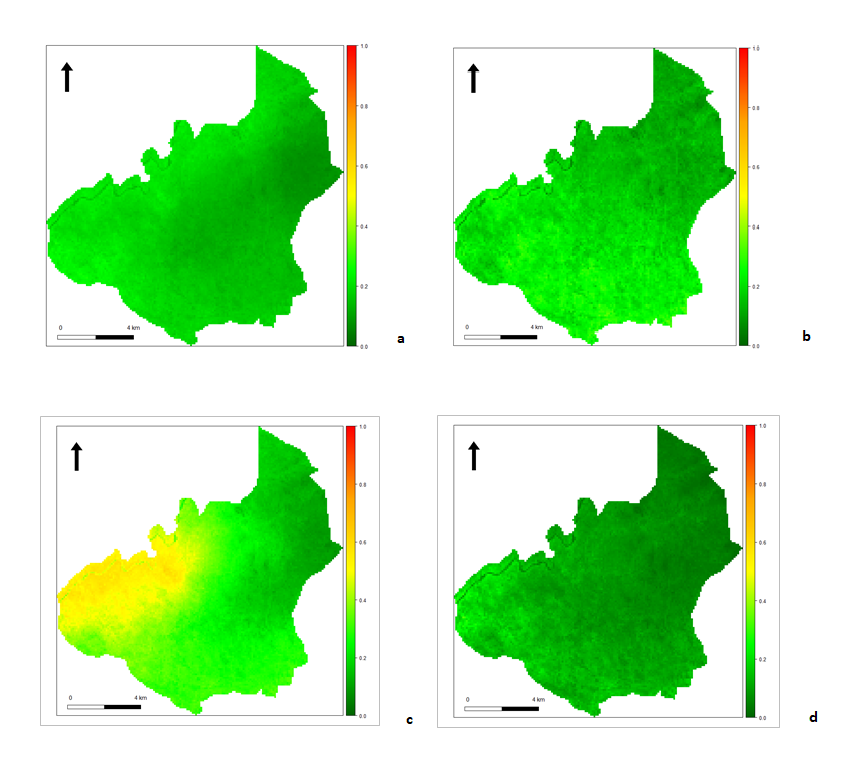

Supplement: Supplementary file 1 — Figure S1. The distribution of malaria incidence by quarter in Focal Area A. a Q1: September to November 2015. b Q2: December 2015 to February 2016. c Q3: March to May 2016. d Q4: June to August 2016. (TIFF 340 kb) [file 13071_2018_2730_MOESM1_ESM.tif]

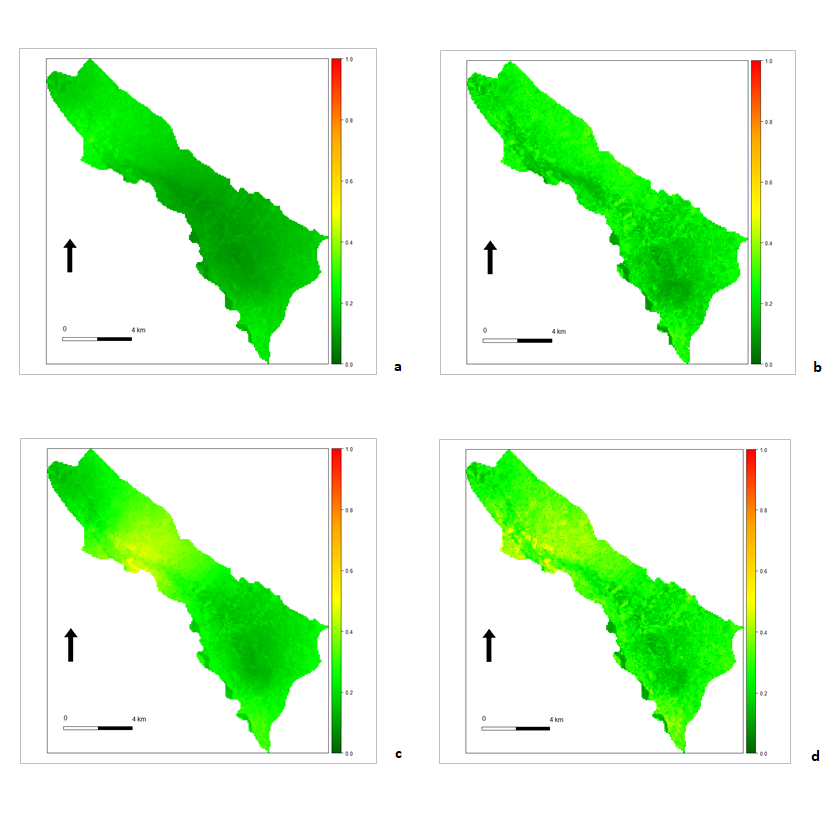

Supplement: Supplementary file 2 — Figure S2. The distribution of malaria incidence by quarter in Focal Area C. a Q1: September to November 2015. b Q2: December 2015 to February 2016. c Q3: March to May 2016. d Q4: June to August 2016. (TIFF 297 kb) [file 13071_2018_2730_MOESM2_ESM.tif]
